# Influence of the Composition, Structure, and Physical and Chemical Properties of Aluminium-Oxide-Based Sorbents on Water Adsorption Ability

**DOI:** 10.3390/ma11010132

**Published:** 2018-01-14

**Authors:** Ruslan Zotov, Eugene Meshcheryakov, Alesia Livanova, Tamara Minakova, Oleg Magaev, Lyubov Isupova, Irina Kurzina

**Affiliations:** 1 Salavat Catalyst Plant, Salavat 453256, Republic of Bashkortostan, Russia; ruslan_zotov@mail.ru; 2 Faculty of Chemistry, National Research Tomsk State University, Tomsk 634050, Russia; meevgeni@mail.ru (E.M.); truelivanova@mail.ru (A.L.); tminakova@mail.tomsknet.ru (T.M.); mov_26@mail.ru (O.M.); 3 Boreskov Institute of Catalysis, Siberian Branch, Russian Academy of Sciences, Novosibirsk 630090, Russia; isupova@catalysis.ru

**Keywords:** aluminium oxide, acid-base properties, kinetics, water adsorption

## Abstract

Interaction between the water adsorption ability of aluminium-oxide-based sorbents and their chemical composition, acid-base properties of the surface, and textural characteristics has been analysed. Alumina desiccants were synthesized with the centrifugal–thermal activation of gibbsite followed by the hydration of the gibbsite under mild conditions. It was demonstrated that the multicyclic adsorption regeneration of samples under realistic conditions results in structural transformations and changes in the acidity of their surfaces and water adsorption ability. The modification of pseudoboehmite with alkali ions increases surface basicity and the dynamic capacity of adsorbents relating to water vapours. Equations have been presented that describe the adsorption and desorption processes taking place during water vapour adsorption with the materials studied.

## 1. Introduction

The use of active alumina as a catalyst in refining processes (e.g., reformation, hydro-treatment, and hydrocracking) and as an adsorbent, in particular, a natural gas desiccant, is widely known. The high activity of aluminium oxide in interaction with polar adsorbates (first, with water vapours) provides deep drying of associated petroleum gas to a dew point of −60 °С and below. Water resistance is an important positive property of aluminium oxide and often determines the selection of aluminium oxide as an adsorbent for drying and the treatment of media containing condensed moisture. The possibility of multiple temperature regeneration by means of coke burn-off ensures the long-lasting work of the adsorbent as a desiccant of olefin-containing flows [1].

Finding new, more effective aluminium-oxide-based adsorbents [2] and the use of an appropriate complex loading of desiccants into adsorbers, which allows for receiving the associated petroleum gas (APG) with the required parameters, remains relevant. It is known, for example, that loading an aluminium oxide protective layer into an adsorber extends the service life of zeolites [3,4]. The use of a more effective desiccant as a protective layer will allow for not only the protection of a bottom layer from condensed moisture but also an enhancement of the effectiveness and operational life of the adsorber.

There are studies on highly effective alumina desiccant adsorbents based on low-temperature forms of aluminium oxide (η-, γ-, and χ-) received by means of the incineration of alkaline hydration products of thermally activated alumina containing bayerite phases of 50% and more [2,5,6]. A characteristic of bayerite-containing hydroxide is the formation of low-temperature phases of aluminium oxide (first, η-modification) at a calcination temperature >300 °С. This provides an opportunity to obtain samples with a developed specific surface area, the required combination of surface sites, a large quantity of micropores, and therefore a larger static capacity than that of the desiccants based on γ-Al_2_O_3_ that are obtained on the basis of pseudoboehmite using the reprecipitation method [5]. The study [6] demonstrated that the dynamic capacity of pseudoboehmite-based desiccants synthesized with the centrifugal–thermal activation of gibbsite with its subsequent hydration under mild conditions is significantly lower than the dynamic capacity of desiccants based on η-Al_2_O_3_. The chemical modification of surfaces with acids and bases has become a more widespread method for increasing the adsorption capacity of active aluminium oxide. A good example is the adsorption capacity of acids or bases, which allows us to modify the concentration of acid–base sites and the reactivity of alumina surface hydroxyls [7].

When sulphuric acid was introduced at the preparation stage of moulding a sorbent mass, the samples received from pseudoboehmite-containing aluminium hydroxide were comparable in dynamic capacity rates to bayerite-based adsorbents, and even exceeded them in static capacity rate [6]. This is related to change in phase composition, textural characteristics, and acid–base surface properties. Moreover, a greater modifying effect was observed in desiccants based on γ-Al_2_O_3_ that had a greater quantity of Bronsted acid sites (BAS) and potent Lewis acid sites (LAS) after the introduction of sulphate ions, and the average diameter of pores was reduced [5]. The dependence of the static capacity value at low relative humidity on the concentration of electron-withdrawing sites has been established. Impregnation with alkali metal hydroxides may also result in the modification and increase of the concentration of surface base sites. Interaction between structural-phase and surface characteristics of adsorbents and their sorption capacity in relation to water is relevant for study.

The purpose of this work is to study the acid–base, texture, and water-absorbing properties of active adsorbents for pseudoboehmite-based samples and for samples modified with alkaline ions.

## 2. Materials and Methods

Samples of bayerite- and pseudoboehmite-based alumina desiccants synthesized with centrifugal–thermal activation gibbsite (CTA GS) followed by its subsequent hydration under mild conditions [6], and also samples of pseudoboehmite-based aluminium oxide modified with sodium and potassium ions, were taken as study objects. The modification was carried out introducing sodium hydroxide and potassium hydroxide solutions at the preparation stage of moulded plastic pastes from pseudoboehmite received by means of the mild hydration of CTA GS product. Adsorbent granules had a diameter of 3.75 ± 0.15 mm at a length of 5 ± 1 mm after the completion of extrusion and the thermal treatment.

A series of studies to determine adsorbent dynamic capacity values on the basis of water vapours under realistic industrial conditions has been carried out for the synthesized samples using a pilot two-reactor adsorption plant (PAP) [8]. Nine (9) adsorption–regeneration cycles were performed for each sample. At set pressure and space velocity values, gas with 100% relative humidity by water had been supplied for drying at an operating temperature of adsorption (22–27 °С): (1) Р = 30 atm; V(N_2_) = 16 m^3^/h; V(Н_2_О) = 11–15 mL/h; (2) Р = 30 atm; V(N_2_) = 10 m^3^/h; and V(Н_2_О) = 7 mL/h. When the dew point temperature (DPT) dropped to −40 °С, the adsorption process was completed and the plant switched automatically to the adsorbent regeneration mode (Р = 25 atm, V(N_2_) = 2 m^3^/h, t 360 min). The final desiccants are characterized by high stability in multiple adsorption–desorption cycles and the possibility of reaching a minimum dew point temperature of −80 °С in the course of drying. Samples modified with alkaline metals are characterized by a greater dynamic capacity compared to the unmodified ones [8].

A series of studies of the physical and chemical properties of the samples obtained was conducted before and after the nine cycles of vapour adsorption–desorption were conducted.

Sodium and potassium contents in samples were determined with inductively coupled plasma mass spectrometry (ICP-MS) using the Agilent 7500cx (Agilent, Santa Clara, CA, USA).

Thermal and gravitational (TG) tests of aluminium oxides were performed in oxidizing medium using the NETZSCH STA 409 synchronous thermal analysis machine. During analysis, the heating rate and exposure time at the selected temperature were varied. Textural characteristics of the CTA GS product and adsorbents were determined by isotherms of nitrogen adsorption at 77 К using the Asap 2400 sorptometer (Micromeritics, Norcross, GA, USA).

Specific surface area was measured using the Brunauer-Emmett-Teller (BET) method, and the micropore volume using the t-method. Mesopore volume was determined by analyzing the integral pore volume distribution curve depending on radius (along adsorption branch); average pore diameter (in nm) was determined with the equation d_ave_ = 4000V_pore_/A, where А is the granule surface area [10].

Determination of the crush strength of samples was carried out using a catalyst strength tester, Lintel PK-21.

The study of acid–base properties of the surfaces of the samples was performed using the pH measurement method in conformity with the technique [11]. The measurement of the pH of the suspension from its formation to the achievement of electrochemical adsorption equilibrium was registered every 5–10 s according to the readings of the IPL-101 ion meter and a рН-meter (рН 673 M) using glass and standard silver chloride electrodes. The values of рН at 5, 10, and 15 s of the sample coming into contact with water and the рН of isoionic state of matter (рНiip), which characterizes the equilibrium state, were selected as the parameters characterizing the acid–base state of the surface.

The dynamic method was used in this study to observe the adsorption of water vapours. The adsorption value was determined by the weight method using a McBenna-Bakr spring scale with a sensitivity of 2.9 × 10^−3^ g/mm. Prior to the performance of the adsorption measurements, each sample was trained (regenerated) at 220–240 °С in a nitrogen flow (extra pure grade nitrogen of impurity content not more than 10 ppm) supplied for 1 h at 5 L/h. Regeneration at temperatures up to 250 °С is considered to be acceptable [12], since it leads to the minimum reduction in the time of useful use of the adsorbent. Wet nitrogen (moisture content >80%) was supplied to the sample to perform adsorption of water vapours. The elongation of the spiral was fixed with a V-630 cathetometer (Instrument-making plant, Kharkiv region, Izyum, Ukraine). All experiments for the study of water vapour kinetics were performed at 26 °С and at atmospheric pressures on the aluminium oxide samples with a fraction of 0.5–1.0 mm. To exclude an impact of the advance speed of a substance on an outer granule surface, a series of experiments on water vapour adsorption at different gradually increasing flow rates up to 36/h had been conducted beforehand. It was found that if the carrier gas velocity was equal to or exceeded 27 L/h, the kinetics of water vapour adsorption did not depend on the nitrogen delivery rate. It was found the optimal adsorbent weighed amount (0.02–0.03 g) allowed us to place adsorbent granules with a fraction of 0.5–1.0 mm in a layer in a foil cup. After the completion of adsorption and the establishment of adsorption balance, dry nitrogen was supplied to the sample at 10 L/h, and the changes in the weight of the sample were recorded at preset time intervals after the water desorption.

## 3. Results

### 3.1. Chemical Composition of Desiccants

According to the ICP-MS results, the sample A-1 contains ~1% of sodium, which is explained by the fact that NaOH was used as an electrolyte at the hydration stage, and potassium traces were also found (Table 1). In the sample A-2, the content of sodium cations is much less, and in the modified samples А-3-Na and А-4-K, the content of the relevant modifying cation is ~2% by weight. The results of the samples before and after nine adsorption–regeneration cycles with the thermogravimetric analysis (TGA) and differential thermal analysis (DTA) methods are given in Table 1. According to the literature [13], the process of water extraction from Al_2_O_3_ is carried out step-by-step. At the first stage (up to 100 °С), the extraction of physically adsorbed water takes place. The extraction of chemically bound water takes place in the second (100–350 °С) and third (350–550 °С) stages. The removal of the residual quantity of water (quantity of hydroxyls remaining on the alumina’s surface equals ~0.1% by weight) takes place at the fourth stage at >550 °С. In the reference sample А-1, one minimum DTA curve, 113 °С, is observed in the area of the extraction of physically adsorbed water, and at a higher temperature (Т = 505 °С), there is a peak that is associated with the degradation of the boehmite phase. According to the thermographs of the samples А-2 and А4-К, water is being extracted already at 52 °С and the maximum extraction level occurs at 136–153 °С. There are no peaks in high-temperature area for these samples.

The thermal and gravitational (TG) and DTA curves for all four samples appeared to be similar after the multicyclic tests. The presence of the peaks at 436–444 °С and 504 °С is observed for these samples on the DTA curve, which is associated with the pseudoboehmite and boehmite degradation, respectively [14]. This fact conforms to the results of the X-ray phase analysis. The assessment after testing showed that up to 5% of the pseudoboehmite phase is formed in samples А-2, А-3-Na, and A-4-K.

### 3.2. Textural Characteristics of Adsorbents

**Figure 1:**
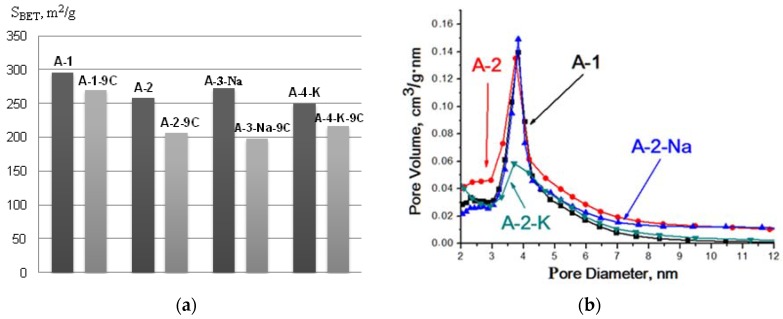

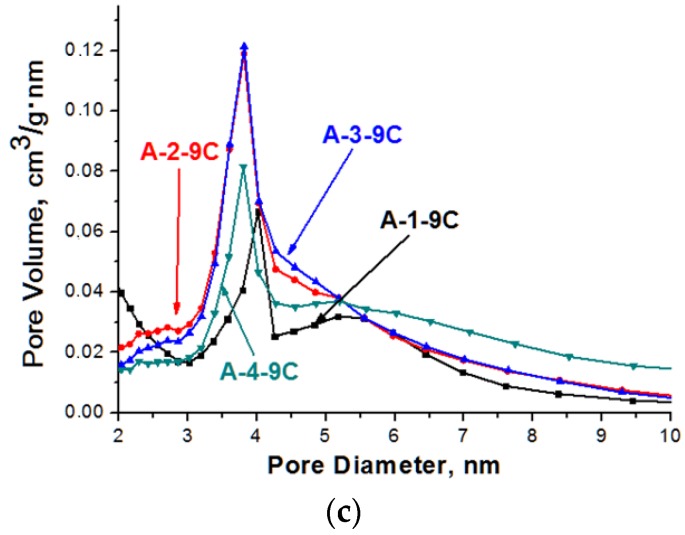
Values of S_BET_ (**а**); pore size distribution of samples А-1, А-2, A-3-Na, and А-4-K (**b**); and А-1-9C, А-2-9C, A-3-Na-9C, and А-4-K-9C (**c**).

All of the samples studied had comparable specific surface areas before testing (within 280–310 m^2^/g). The average adsorbent particle size is 100 nm. After testing (nine adsorption−desorption cycles), the specific surface area reduces, and this becomes especially noticeable for the sample А-2 (308 to 201 m^2^/g) (Table 2, Figure 1).

The pore volume for sample А-1 had the least value (0.27 cm^3^/g) at the pore diameter of 3.3 nm, and it was reduced even more after the completion of the sorption−regeneration cycles. The pore volume was slightly higher for the sample А-2 (0.32 cm^3^/g) at the pore diameter of 3.7 nm and it also was a bit smaller after testing. The pore volume for the samples А-3-Na and А-4-К modified with Na and K was >~0.40 cm^3^/g at the pore diameter of 5.0 and 4.9 nm, respectively. After the cycle tests of water adsorption–desorption, the pore volume remained nearly the same in these samples, while the diameters increased slightly to 6.3 and 6.2 nm, respectively.

The pore distribution by size for each of the desiccant samples is given in Figure 1. The original samples are characterized by the presence of fine mesopores in the structure, which are in the narrow distribution interval of 3.0–4.5 nm. After the multicyclic tests, the share of fine mesopores decreased and larger pores of 4.5–7.0 nm appeared. A bimodal distribution of the second in the interval of 5–7 nm was observed for the samples (A-1 and A-4-К) after nine cycles were completed. The monitored changes in porous desiccant structures could be conditioned by the sintering/“healing” processes of the smallest pores during regeneration.

Mechanical and physical properties of the samples were studied. It was demonstrated that the bulk density of the samples А-1 and А-2 was 0.87 g/cm^3^ and 0.83 g/cm^3^, respectively. The modified aluminium oxides are 0.73–0.74 g/cm^3^. The crush strength of the non-modified samples А-1 and А-2 (Table 2) was slightly higher compared to the samples modified with Na and K. After the cycle tests, the crush strength of the samples did not change within the range of the calculated error. The crush strength of the aluminium oxide samples studied was comparable to the strength of industrial zeolites of the KA type used for APG drying.

### 3.3. Acid–Base Properties of Alumina Desiccants

The moisture adsorption and water vapour adsorption are determined by the acid–base properties of the surface. It is known that pH change in the aqueous suspension of the sample within a period of time proceeds for each individual substance with an intrinsic trend of kinetic curves and with their different arrangement relative to the acidity level of the original electrolyte (pH_0_) [15]. The acid–base parameter (рН of isoionic state of matter (рНiip)) had been determined from the curves, showing the dependence of the рН of the desiccant samples’ aqueous suspension on the period of time after the establishment of the balance. The parameter corresponds to the рН value at which, when different ions are available in solution, the equal adsorption of acid and base groups is established on the solid body surface. This parameter characterizes the relative content of acid and base sites on the solid body surface.

**Figure 2:**
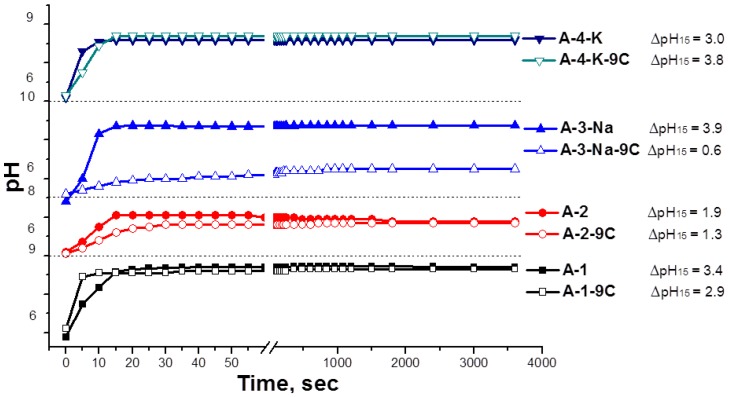
Kinetic curves of the pH of the aqueous suspensions of the initial samples’ dryers and dehumidifiers after nine cycles of adsorption–regeneration, ΔрН_15_: change in pH of the sample for 15 s of contact with water.

The kinetic curves of the pH change of the original samples and samples after nine water adsorption and desorption cycles for each sample are given in Figure 2. It can be observed that the differences in the studied samples by suspension basicity are manifested already during the first seconds of contact. The arrangement of kinetic curves for all of the samples is above the neutrality level, and abrupt alkalizing at initial time, an increase of рН_10″_, рН_15″_ values, and the higher rate of рН change (Figure 2) indicate the presence of potent base sites that are aprotic on their surfaces.

The рН value of the suspension when there is longer contact with the aqueous medium reflects the slowly proceeding processes of dilution, hydration, and hydrolysis of aluminium cations with further establishment of acid−base equilibrium in the system and is characterized by the рНiit value. The tests demonstrate that an elongation of the period of contact of the samples of Al_2_O_3_ with water to 1 h and longer does not lead to further change in the pH of the suspension. The wide plateau at 50–250 s at considerably high pH values proves the prevalence of potent Bronsted sites of base nature on the aluminium oxide’s surface.

### 3.4. Kinetics of Water Vapour Adsorption on Alumina Desiccants

The water adsorption dependence on the structure and strength of surface sites may correspond to different kinetic parameters. The kinetic interaction parameters with water vapours are needed to calculate the hydrodynamic parameters of industrial adsorbents and the optimal granule shapes of alumina adsorbents. The adsorbate saturation of each adsorbent particle in the adsorber depends on the diffusion rate of the absorbed molecules inside of the granules, which finally determines the mass transfer rate under a particular hydrodynamic mode. The kinetics of water vapor were studied to estimate the rate of change in the adsorption capacity of the granules in single time. Studies of water vapour kinetics were conducted to assess the rate of change with time in the adsorption capacity of single granules.

**Figure 3:**
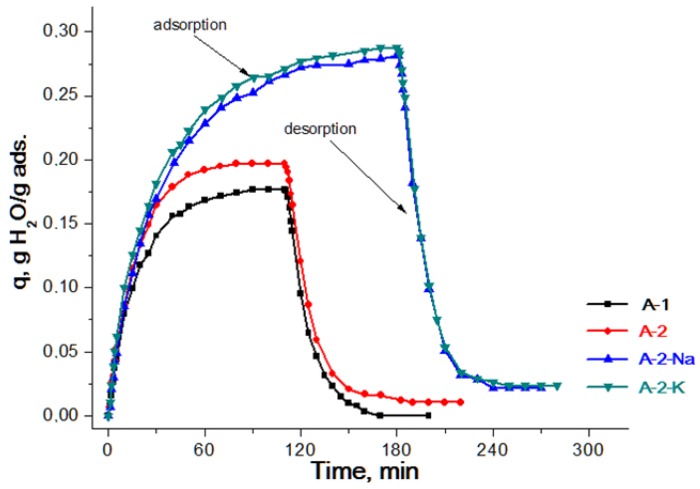
Kinetic curves of the adsorption and desorption of water vapor on aluminum oxide samples for a fraction of 0.5–1.0 mm (conditions: carrier gas adsorption rate at 30 L/h, desorption at 10 L/h).

From the kinetic curves of water vapour adsorption on samples А-1, А-2, A-3-Na, and А-4-K given in Figure 3, one can see that the adsorption capacity by water vapours under the given conditions is higher for the samples А-3-Na and А-4-K modified with sodium and potassium ions compared to the original sample А-2 and the bayerite-based sample (А-1). Moreover, the kinetic curves of the samples with admixtures have only a slight difference. Unlike the unmodified samples, these adsorbents are characterized by an irreversibility effect: the curves do not come to the initial state after desorption (the difference is ~8% by weight). This could be explained by the presence of the Lewis base sites on the surface of these compounds, which are capable of strongly adsorbing water. During the repeated tests, water interaction with the surface passes through the formation of hydrogen bonds and the process becomes reversible. It was proven for the sample А-4-K that no changes were observed in the trend of kinetic curves for a training period elongation of the sample to 2 h and a temperature increase to 250 °С.

**Figure 4:**
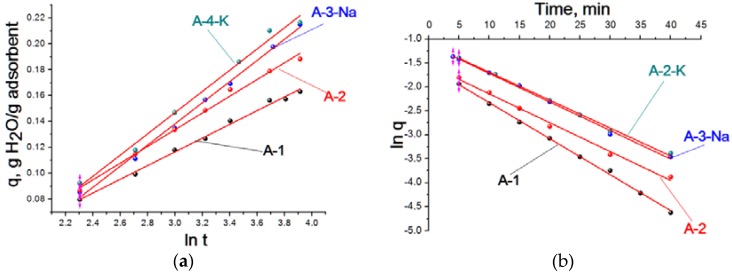
Approximations of the kinetic curves sorption of water vapor on the samples A-1, А-2, A-3-Na, and A-2-K: (**a**) the value of adsorption (q): logarithm of time (ln t); (**b**) logarithm of value of adsorption (ln q) time (t).

Various kinetic models were considered for the mathematical description of the kinetic curves [16,17]. It was established that in the region of a surface coverage ratio of >0.4–0.6, the kinetic adsorption curves of every sample are rectified (Figure 4) in the coordinates of the adsorption value (a) and the logarithm of time (ln t). In this article, we used the method of least squares with a correlation coefficient of 0.993–0.992. The experimental data are rectified in the given coordinates, when the adsorption kinetics are expressed by the Roginsky–Zeldovich equation:

q = B ln t + C
(1)

where a is the amount of adsorbed gas and B and C are constants.

The Roginsky–Zeldovich equation is typical for those processes run on proportionally non-uniform surfaces, and also for multicenter chemosorption on a uniform surface in a certain region of the average surface coverages. The factor B was found, confirming the nature of adsorbate interaction with the adsorbent surface (Table 3). Increase in the factor B was observed for the modified samples, which is associated with the growth of interaction energy between adsorbate and adsorbent and, therefore, with the availability of potent surface base sites.

The theoretical fundamentals of the desorption stage are developed poorly in comparison with the kinetics theory and even adsorption dynamics, so the majority of research proposes empirical decisions for the special cases containing a number of assumptions [1]. Since the external mass transfer does not have a significant impact on the processes overall at relatively high gas flow rates, and the kinetic desorption curves received in the course of gas purging tests are close to the kinetic curves in vacuum, then it is possible to apply the equation proposed in the study [1]:

ln (q/q_max_) = −k × t (2)

where q_max_ is the maximum quantity of the adsorbed substance and k is a factor (adsorption rate).

According to the theory, the factor k is proportional to exp (Ed/RT), where T is the temperature in K, R is the ideal-gas constant. Ed is the activation energy of the desorption process bound with the adsorption heat with the equation:

Ed = Q + Ea
(3)

where Q is the adsorption heat and Ea is the adsorption activation energy.

The desorption kinetics curves in the region of the average coverages are rectified in the coordinates ln q−t (Figure 4). The calculated factors В and K given in Table 3 show that the factors В and K as well as the maximum adsorption value for the samples А-3-Na and А-4-K are close to each other. Factor B is increased in the line of А-1, А-2, А-4-K, and А-3-Na, and the factor k is decreased, which confirms an improvement in the bonding strength of adsorbed molecules with the surface. The maximum value of water vapour adsorption increases proportionally for the samples received in the course of study of the water vapour adsorption kinetics.

## 4. Discussion

A comprehensive study of desiccants based on aluminium oxide modified with alkaline metals, including their physicochemical, mechanical, and physical properties and water vapour adsorption kinetics, has been conducted. The samples were studied before and after nine water adsorption–desorption cycles under maximally realistic conditions to the industrial parameters during the drying and transportation of the associated petroleum gas. It was found that the samples of bayerite- and pseudoboehmite-based alumina desiccants have potent base sites on their surface. The dynamic capacity of adsorbents with respect to water vapours increases if modified with alkali ions against the background of an enlargement of the average diameter and pore volume. This is associated with the growth of the concentration and strength of surface base sites due to the effect of alkaline cations [18].

As soon as these samples were used in the nine adsorption–regeneration cycles at the pilot adsorption plant under the pressure of 30 atm, the phase composition of samples А-2, А-3-Na, and А-4-K underwent minor changes: an additional pseudoboehmite phase, a pore size enlargement, a decrease in fine pore quantity, and a reduction in the specific surface area. The specific surface area of the samples А-2 and А-3-Na is reduced more intensively and an increase in the surface acidity of the samples was observed after nine adsorption–regeneration cycles. The value of pHiit for the samples А-1 and А-4-K before and after their use as desiccants coincides, i.e., the total surface basicity did not change. It can be noted that quite a high stability of these samples was observed during the adsorption–regeneration cycles at the pilot plant.

The testing of the sample with the TGA and DTA methods demonstrated that the peak displacements of physically adsorbed water to a lower temperature region (81–86 °С) were observed for the samples used in multicyclic drying with an increase in the volume of water removed at temperatures up to 240 °С. A greater amount of water released at these temperatures was observed in the modified samples А-3-Na-9C and А-4-K-9C having a larger volume and pore diameter, which confirms the larger water capacity of these samples.

The adsorption kinetics of all the samples in the region of average coverage are expressed by the Roginsky–Zeldovich equation, which characterizes adsorption with a proportionally non-uniform surface. The desorption is carried out from the uniform surface. The water vapour adsorption on active aluminium oxide is the combined result of three processes: chemosorption, physical adsorption, and capillary condensation. As follows from the data, the first layer is formed on active surface sites of aluminium oxide due to dissociation chemosorption of water molecules. The second and the next layers are adsorbed physically by means of van der Waals strength. The capillary condensation at a relative vapour pressure below one P/P_0_ and at a temperature above the dew point of the liquid is typical for this adsorbent. The factors of the adsorption and desorption equations calculated are connected with the binding energy of the surface sites’ water adsorption. There were higher adsorption factor values set in the Roginsky–Zeldovich equation for aluminium oxides modified with alkali metal cations.

## 5. Conclusions

As a result of the research, it was found that the modification of an alumina-based desiccant by alkaline cations makes it possible to increase its efficiency during the adsorption of water vapor. As a result of alkaline modification, a decrease in the specific surface area and an increase in the average pore diameter were observed. The study of the kinetics of the adsorption of water vapor showed that the highest adsorption rate is characteristic of adsorbents A-1 and A-2. Despite this fact, a high adsorption capacity (~0.28 g/g ads) was characteristic of the modified samples. It was revealed that the multicyclic pressure tests do not significantly affect the functional characteristics of the alumina adsorbent samples.

## Figures and Tables

**Table 1:** DTA and TGA data of the alumina samples.

Sample	The Weight Loss on the TG Curve, %				Тmax (DTA), °С	ТPT (DTA), °С
	To 240 °С (Δm1)	From 240 °С to 350 °С (Δm2)	From 350 °С to 550 °С (Δm3)	From 550 °С to 900 °С (Δm4)		
А-1	4.8	1.0	2.2	2.0	113	505
А-2	2.5	1.0	1.3	1.9	(52) 140	–
А-3-Na	2.3	0.9	1.0	1.6	(52) 153	–
А-4-K	2.5	0.9	0.9	1.8	(52) 136	–
А-1-9C	6.9	0.8	3.0	1.7	86	504
А-2-9C	5.3	0.9	2.9	1.4	85	436
А-3-Na-9C	9.1	1.0	2.5	1.3	81	445
А-4-K-9C	10.6	0.8	2.4	1.3	81	444

Δm1—weight change determined by TGA at a heating rate of 10°/min to 240 °С integration; ∆m2—weight change determined by TGA at a heating rate of 2°/min to 350 °C and holding 1 h; Δm3—weight change determined by TGA at a heating rate of 10°/min to 900 °С integration. TG: thermal and gravitational. ТPT: The Phase Transition Temperature.

**Table 2:** Texture characteristics, the crush strength values, and modifications to the content of additives in the alumina samples before and after pressure tests.

Сharacteristics	А-1	А-2	А-3-Na	А-4-K	А-1-9C	А-2-9C	А-3-Na-9C	А-4-K-9C
S_BET_ ± Δ, m^2^/g	279 ± 27	308 ± 30	312 ± 31	288 ± 28	265 ± 26	201 ± 20	230 ± 23	233 ± 23
The crush strength, MPa	25	25	13	17	23	24	14	13
Content of modifying additives, % mass.	0.98 Na; 0.04 К	0.12 Na; 0.02 К	2.10 Na; 0.03 К	0.11 Na; 2.20 K	0.97 Na; 0.04 К	0.11 Na; 0.01 К	2.00 Na; 0.02 К	0.13 Na; 2.10 K

**Table 3:** Characteristics of the kinetic curves of the adsorption and desorption of water vapor on the alumina samples.

Sample	q_m_, g/g ads	B	k
А-1	0.177	0.053	0.0752
А-2	0.197	0.063	0.0603
А-3-Na	0.281	0.086	0.0596
А-4-K	0.281	0.085	0.0583

q_m_: the maximum amount of water vapor adsorbed on the sample; B and k: coefficients of approximating the kinetic curves of adsorption and desorption, respectively.
